# Functional disability and cognition in relation to residential transitions in older adults: Evidence from three French cohorts, 1988-2018

**DOI:** 10.1016/j.tjfa.2026.100159

**Published:** 2026-05-24

**Authors:** Jeanne Bardinet, Luc Letenneur, Alice Pellichero, Denis Boucaud-Maitre, Maturin Tabue-Teguo, Hélène Amieva, Karine Pérès

**Affiliations:** a Univ. Bordeaux, INSERM, BPH, U1219, F-33000 Bordeaux, France; b Centre Hospitalier le Vinatier, Direction de la Recherche et de l’Innovation, Bron, France; c University of Antilles, EPICLIV, Fort-de-France, Martinique

**Keywords:** Functional disability, ADL, IADL, Cognition, Older adults, Residential transition

## Abstract

**Background:**

Aging is often accompanied by increasing difficulties in daily living that may compromise aging in place. In France, three main housing types can be distinguished: ordinary home (OH), intermediate housing (IH) and nursing home (NH). This study aimed to explore the relationship between functional disability, cognition, and residential transitions in older adults.

**Methods:**

Participants aged 65 years and older living in OH at baseline were drawn from three French population-based cohorts (PAQUID, Three-City and AMI), followed for up to 30 years. A hierarchical indicator of functional disability was derived from mobility, IADL and ADL disabilities, while cognitive function was assessed using the Mini Mental State Examination (MMSE). A multi-state model was applied to investigate the associations between functional disability and cognition (as time-dependent variables) with housing transitions, adjusting for sex, cohort, marital status and education. We also examined the involvement of IADL disabilities on transitions from OH to IH or NH in sex-stratified models. Sensitivity analyses were performed using lagged measures.

**Results:**

Among 5264 included participants (mean age 74.7 ± 6.0 years; 56.9% women), 832 relocated from OH during a median follow-up of 10.2 years, including 216 to IH and 616 to NH. Mild and moderate disability, as lower MMSE scores, were independently associated with higher risks of transitioning to IH or NH, while severe disability and cognitive impairment were only associated with NH transitions. In sex-stratified analyses, transportation difficulties were associated with IH and NH transitions among women, whereas only with NH admission in men. Difficulties with handling finances in women, with medications management in men, and difficulties with shopping in both sexes were also associated with transition to NH.

**Discussion:**

Intermediate housing was more frequently associated with mild or moderate disability, whereas transitions to long-term care were more often observed in participants with severe cognitive decline.

## Highlights

• Living at home is an individual and societal priority, but it is not possible for everyone until the end of life.
• Transitioning to intermediate housing (IH) is less likely than transitioning to nursing homes.
• Transitioning to IH is more likely among older adults with mild or moderate disability or severe cognitive impairments.
• Severe cognitive decline with major impact on daily living strongly predicts nursing home entry.
• Sex-specific patterns in activity limitations highlight the need for tailored support strategies.

## Graphical abstract

**Figure:**
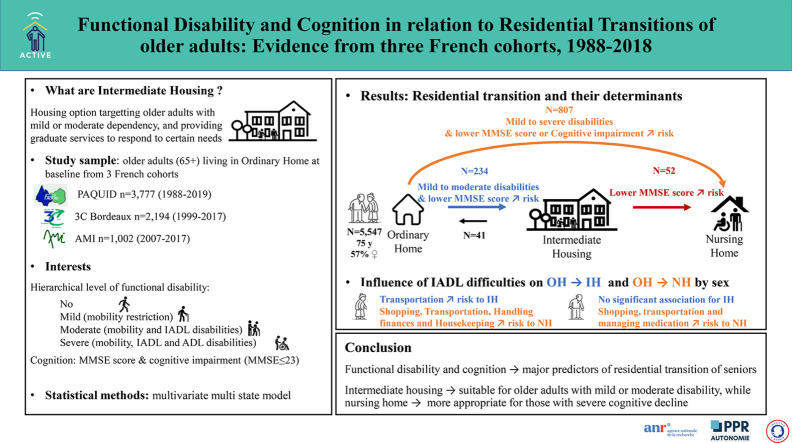

## 1 Introduction

Population aging represents a major public health concern affecting demographic structures, healthcare systems and family dynamics [1,2]. At the individual level, aging often leads to functional and cognitive decline, requiring either technical and home adaptations, professional and familial assistance or relocation to more suitable accommodations [3,4]. Housing options for older adults are generally classified into three levels: ordinary housing (OH), intermediate housing (IH), and long term care facilities such as nursing home (NH) [5,6]. While OH may not always be suited for older adults with disability, it can be adapted to accommodate their needs [7,8]. IH refers to housing arrangements positioned between OH for fully independent individuals, and medicalized long-term care facilities (NH) for those with severe disabilities. IH includes senior housing or residential care facilities and targets individuals with mild to moderate dependency by providing a safe and supportive environment, offering tiered services, ranging from social gathering spaces aimed at reducing social isolation to household assistance, safety features, daily support services, and organized activities [9,10]. In France, IH may include publicly or privately managed senior residences. NH, by contrast, are designed for individuals with severe dependency combining disability in basic and instrumental activities of daily living (ADL/IADL), often associated with cognitive impairment and dementia. Although the demand for medicalized care is expected to rise rapidly and exceed short-term capacity [11], about 90% of the older adults want to age and live in place [12,13]. Longitudinal studies consistently establish that functional disabilities develop progressively with age [14,15], and IADL disabilities are recognized as early markers of functional and cognitive decline [16,17]. Preventing early person-environment mismatches thus becomes essential [7], and IH could represent a key alternative. However, the conditions under which it becomes a preferred or viable option remain unclear. Although the literature has consistently identified global functional disability and cognitive impairment as major predictors of institutionalization [18,19], much less is known about transitions to IH. Indeed, functional decline is a well-established risk factor for relocation to either IH or NH [[20], [21], [22], [23]], but few studies have explored the specific daily living difficulties that drive these transitions, and most existing evidence relies on small or selective samples with short follow-ups [16,24]. A better understanding of the determinants leading to residential transitions is essential for better anticipating, designing and planning future accommodations for the older population.

Using data from three French population-based cohorts of older adults followed for over 30 years, this study aims to describe residential transitions among adults aged 65 years and older and to examine the associations between functional disability and cognition with these trajectories, with particular attention to potential sex differences regarding specific IADL disability.

## 2 Methods

### 2.1 Source population

Data were drawn from three French population-based cohorts of older adults originally designed to investigate cerebral and functional aging: PAQUID (1988–2018), Three-City (3C) (1999–2017) and Aging Multidisciplinary Investigation (AMI) (2007–2017) [[25], [26], [27]]. All studies were approved by the Ethics Committee of the Bordeaux University Hospital and participants provided written informed consent. Participants were followed every two to three years through standardized interviews collecting sociodemographic, lifestyle and health information. Comparable designs and assessment tools across cohorts enabled pooled analyses. The long follow-up of the PAQUID cohort (1988–2018) overlaps with the observation periods of the 3C (1999–2017) and AMI (2007–2017) cohorts, providing complementary temporal coverage of older populations across the study period. Variables were harmonized across cohorts using common definitions to ensure comparability of measures over time. The protocols and methodologies of the three cohorts have been described in detail elsewhere [[25], [26], [27]].

### 2.2 Study sample

Eligible sample comprised all participants aged 65 years and older, living in OH at baseline, with available information on housing status for at least two follow-up visits. The analytic sample excluded participants with missing data on functional disability or confounders.

### 2.3 Functional disability

Functional disability was assessed at each follow-up visit by trained psychologists using validated instruments. In the present study, we focused on the three domains of disability. Mobility was evaluated using the three items of the Rosow and Breslau scale [28]: perform heavy housework; climb stairs to the second floor; and walk half a mile. IADL disability was assessed using the Lawton scale [29], including five items for men, and eight for women (use the telephone, do the shopping, use transportation, manage medications and handle finances; for women, additional items assess meal preparation, housekeeping and laundry), reflecting gender roles in the generations studied. ADL disability was measured using the Katz scale [30], through five items: bathing, dressing, toileting, transferring and feeding. For each scale, disability was defined when participants were unable to perform at least one activity of the scale without human assistance.

As previously published [31], a hierarchical indicator of functional disability was constructed: 1) no disability, 2) mild disability (only mobility limitation), 3) moderate disability (mobility and IADL disabilities), and 4) severe disability (mobility, IADL and ADL disabilities).

### 2.4 Cognition

Cognitive function was evaluated using the Mini-Mental State Examination (MMSE, range 0–30; higher scores indicate better performance) [32]. Cognitive impairment was defined as MMSE score≤23/30, as proposed by Ruchinskas and Curyto [33].

### 2.5 Housing and residential transitions

At each follow-up, participants reported their current housing, categorized as OH (personal or family home), IH (senior housing and rural collective home for older adults) and NH (long-term or medicalized care facilities). Residential transitions were defined as changes between OH, IH and NH, between two consecutive visits. Because transitions were recorded as discrete intervals, timing was treated as interval-censored.

### 2.6 Other variables

Sociodemographic characteristics included age, sex, educational level (no formal education or primary school, secondary school, or high school and above), living area (rural for less than 2000 inhabitants vs urban), and marital status (single or separated, in a union, or widowed). Medical characteristics included the number of medications (based on medical prescriptions), visual impairment (defined as a Parinaud score >2 [34] or self-reported), hearing impairment (self-reported), a clinical diagnosis of dementia, depressive symptomatology using the Center for Epidemiological Studies-Depression (CES-D) scale (with a threshold of ≥17 for men and ≥23 for women [35]) and loneliness feeling (by the item 14 of the CES-D scale).

### 2.7 Statistical analysis

#### 2.7.1 Descriptive analyses

Baseline characteristics were described overall and by sex using Chi-square tests and Student’s t-tests as appropriate. Then, a descriptive overview of the follow-up data was conducted, including the number and types of residential transitions observed among participants from both the eligible and the analytic sample.

#### 2.7.2 Associations between disability and cognition with residential transitions

Multi-state models were applied to describe residential transitions and to estimate the associations between time-varying functional disability and cognition with housing trajectories, using the *msm* package in R [36,37]. All the assumptions of the model were checked, including linearity of quantitative variable using natural cubic splines and inspection of model fit. The model included four states (OH, IH, NH, and death) and accounted for interval censoring, time-dependent covariates, and the competing risk of death. Allowed transitions were OH to IH, OH to NH, OH to death, IH to OH, IH to NH, IH to death, and NH to death; rare reverse transitions from NH to other housing were censored (<0.01% of all transitions). Participants were censored at the date of their last completed follow-up visit if they were lost to follow-up. Participants who missed an intermediate visit but returned later remained in the risk set, and their time-dependent covariates were updated at the next observed visit. The corresponding transition intensity matrix (Q-matrix) and mean sojourn time in each housing are provided for eligible and analytic sample in Supplementary **Table S1**. The model assumes a continuous-time, time-homogeneous Markov process, whereby transition intensities depend only on the current state and covariate values. Age was used as the underlying time scale and age-specific state occupancy probabilities derived from the estimated Q-matrix, were estimated using the *pmatrix.msm* function at ages 70, 80, and 90 years. These represent model-based probabilities at those ages rather than probabilities over a fixed follow-up period.

A multivariate model then examined the effects of functional disability (four-level hierarchical indicator) and cognition (MMSE score) on residential transitions, adjusting for sex, cohort, marital status, and education. Functional disability and cognition were included as time-varying covariates measured at the visit when the transition was detected (T), acknowledging that the transition occurred between the previous (T − 1) and current (T) visits. Sensitivity analyses using lagged covariates measured at T − 1 were conducted to assess potential reverse causality. Contextual variables more susceptible to reverse causation (e.g., marital status) were systematically defined at T − 1 to ensure temporal ordering. To assess potential confounding by health status, an additional sensitivity analysis was performed by further adjusting the main model for polypharmacy (≥6 medications).

To disentangle the combined association of cognitive impairment and IADL disability, cognitive impairment (MMSE score ≤23/30 [33]) was substituted for the continuous MMSE score, and a composite four-category variable was created: (1) no IADL disability and no cognitive impairment; (2) IADL disability without cognitive impairment; (3) cognitive impairment without IADL disability; and (4) both IADL disability and cognitive impairment.

#### 2.7.3 Focus on IADL disability in sex-stratified analysis

Finally, a sex-stratified multi-state model was fitted to explore sex-specific associations between individual IADL disability and residential transitions. For this analysis, participants without at least two complete follow-up visits for IADL disability were excluded, and allowed transitions were limited to OH to IH, OH to NH, and OH to Death, due to the small number of men or women transitioning from IH to OH or IH to NH. As in the main model, IADL disability and cognition were defined at the visit when the transition was detected (time T), with sensitivity analyses using values measured at the preceding wave (T − 1).

Hazard ratios (HRs) and 95% confidence intervals (CIs) were reported, with statistical significance was set at p-value <0.05. All analyses were performed using R Software (R version 4.5.0).

## 3 Results

### 3.1 Study sample description

**Fig. 1:**
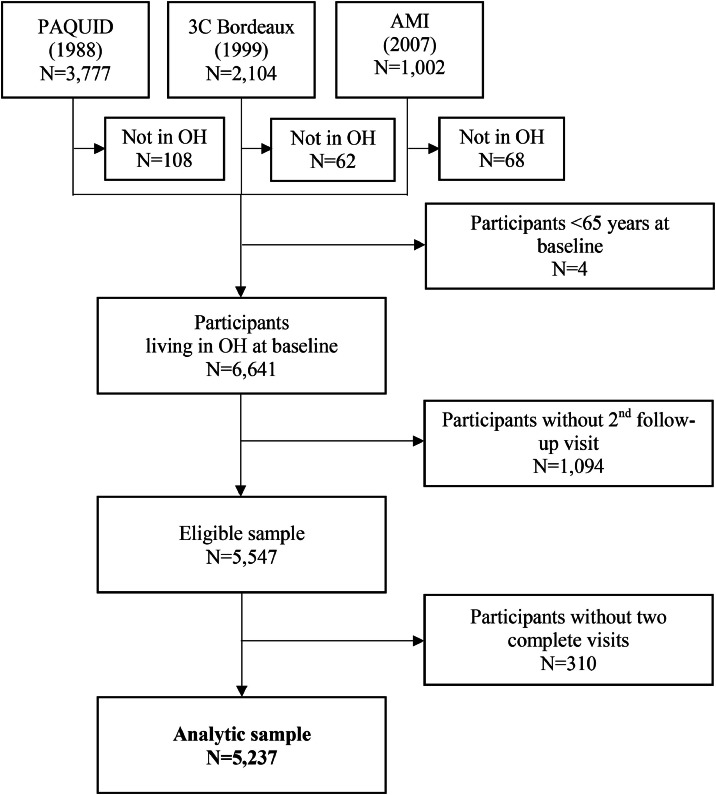
Flow chart, study of functional disability, cognition, and residential transitions of older adults, from PAQUID, 3-City and AMI cohorts, 1988, 1999, 2007.

After eligibility criteria, 5237 participants were included in the overall analysis (Fig. 1). Baseline characteristics are presented in Table 1. Participants were predominantly women (57.0%), with a mean age of 74.7 (± 6.0) years; 68% lived in urban area and 60% were in a union. At baseline, 43% had mild disability, 17% moderate and 2% severe disability. Compared with men, women were older, more often widowed, less educated, and more frequently reported depressive symptoms and loneliness.

**Table 1:** Description of sociodemographic and health characteristics of the overall study sample and according to the sex, from PAQUID (1988), 3-City (1999) and AMI (2007) cohorts at baseline (n = 5237).

	Overall (n = 5237)	Men (n = 2254)	Women (n = 2983)	p-value¹
	N (%) / Mean ± SD	N (%) / Mean ± SD	N (%) / Mean ± SD	
Mean age (years)	74.7 ± 6.0	74.3 ± 5.8	75.0 ± 6.1	<0.001
Living in urban area	3573 (68.2%)	1412 (62.6%)	2161 (72.4%)	<0.001
Cohort				<0.001
PAQUID (1989)	2787 (53.2%)	1164 (51.6%)	1623 (54.4%)	
3-City (1999)	680 (13.0%)	411 (18.2%)	269 (9.0%)	
AMI (2007)	1770 (33.8%)	679 (30.1%)	1091 (36.6%)	
Marital status				<0.001
Alone	250 (4.8%)	64 (2.8%)	186 (6.2%)	
In union	3134 (59.8%)	1856 (82.3%)	1278 (42.8%)	
Widowed	1623 (31.0%)	269 (11.9%)	1354 (45.4%)	
Separated	230 (4.4%)	65 (2.9%)	165 (5.5%)	
Educational level				<0.001
Low	1439 (27.5%)	578 (25.6%)	861 (28.9%)	
Intermediate	1871 (35.7%)	775 (34.4%)	1096 (36.7%)	
High	1927 (36.8%)	901 (40.0%)	1026 (34.4%)	
Mean number of medication	4.3 ± 2.8	3.9 ± 2.8	4.5 ± 2.8	<0.001
Visual impairment	1264 (24.4%)	542 (24.4%)	722 (24.4%)	>0.900
Hearing impairment	1144 (22.0%)	592 (26.5%)	552 (18.7%)	<0.001
Dementia (diagnosis)	114 (2.2%)	53 (2.3%)	61 (2.0%)	0.500
Mean MMSE score	26.4 ± 3.1	26.6 ± 3.0	26.2 ± 3.2	<0.001
Depressive symptomatology*	504 (9.8%)	173 (7.9%)	331 (11.3%)	<0.001
Feeling of loneliness	1293 (25.2%)	297 (13.5%)	996 (34.0%)	<0.001
Hierarchical indicator of disability				<0.001
No disability	2003 (38.2%)	1103 (48.9%)	900 (30.1%)	
Mild	2265 (43.2%)	861 (38.2%)	1404 (47.1%)	
Moderate	876 (16.7%)	257 (11.4%)	619 (20.7%)	
Severe	93 (1.8%)	33 (1.5%)	60 (2.0%)	
Mean time of follow-up (in years)	13 ± 7	12 ± 6	14 ± 7	<0.001

CES-D: Center for Epidemiologic Studies-Depression; MMSE: Mini Mental State Examination

¹p-value from Chi-square tests for qualitative variables and from Student t-tests for quantitative variables.

*Depressive symptomatology: CES-D score≥17 for men and ≥23 for women.

### 3.2 Residential transitions

**Fig. 2:**
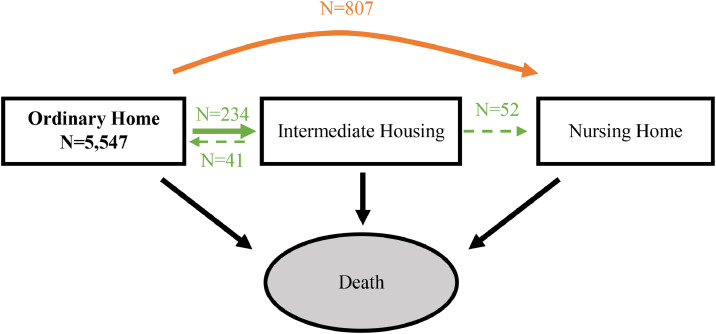
Residential transitions of eligible sample of older adults living in OH, from PAQUID, 3C and AMI cohorts, 1988–2018 (n = 5 547).

From the eligible study sample (N = 5547 individuals, all living in OH at baseline), over a median follow-up of 14.9 years (range 1–35 years), 1041 participants (18.7%) experienced a residential transition from OH: 807 (77.5%) moved to NH and 234 (22.5%) to IH. Among those moving from OH to IH, 17% subsequently returned to OH, and 22% subsequently entered NH (Fig. 2). In the eligible sample, the probabilities of remaining in OH decreased steadily with age for both sexes, with transitions to NH more frequent than those to IH (Table 2). For example, at age 70, probabilities of remaining in OH, moving to IH, moving to NH or dying were 0.49, 0.02, 0.05 and 0.44 in women vs 0.46, 0.01, 0.01 and 0.53 in men. At age 90, these values attained 0.12, 0.00, 0.01 and 0.86 in women and 0.09, 0.00, 0.01 and 0.90 in men, respectively.

**Table 2:** Probability of residential transitions with 95% confidence intervals for men and women at 70, 80 and 90 years*, based on the PAQUID (1988–2018), 3-City (1999–2018) and AMI (2007–2017) cohorts (N = 5547).

	Women (N = 3150)				Men (N = 2397)			
To	OH	IH	NH	Death	OH	IH	NH	Death
**From**								
At 70 years								
**OH**	0.49 [0.48–0.50]	0.02 [0.01–0.02]	0.05 [0.04–0.05]	0.44 [0.43–0.46]	0.46 [0.44–0.47]	0.01 [0.00–0.01]	0.01 [0.01–0.02]	0.53 [0.51–0.54]
**IH**	0.12 [0.09–0.17]	0.08 [0.05–0.12]	0.07 [0.05–0.09]	0.72 [0.67–0.77]	0.12 [0.06–0.20]	0.06 [0.02–0.12]	0.03 [0.01–0.05]	0.79 [0.70–0.88]
**NH**	-	-	0.05 [0.04–0.07]	0.95 [0.93–0.96]	-	-	0.02 [0.01–0.03]	0.98 [0.97–0.99]
At 80 years								
**OH**	0.24 [0.23–0.26]	0.01 [0.01–0.01]	0.03 [0.02–0.03]	0.72 [0.71–0.73]	0.20 [0.19–0.22]	0.00 [0.00–0.00]	0.01 [0.00–0.01]	0.78 [0.77–0.80]
**IH**	0.07 [0.05–0.10]	0.01 [0.00–0.02]	0.02 [0.01–0.02]	0.90 [0.87–0.93]	0.06 [0.03–0.11]	0.00 [0.00–0.02]	0.00 [0.00–0.01]	0.93 [0.88–0.97]
**NH**	-	-	0.00 [0.00–0.00]	1.00 [0.99–1.00]	-	-	0.00 [0.00–0.00]	1.00 [1.00–1.00]
At 90 years								
**OH**	0.12 [0.11–0.13]	0.00 [0.00–0.01]	0.01 [0.01–0.01]	0.86 [0.85–0.87]	0.09 [0.08–0.10]	0.00 [0.00–0.00]	0.00 [0.00–0.00]	0.90 [0.89–0.91]
**IH**	0.04 [0.02–0.05]	0.00 [0.00–0.00]	0.00 [0.00–0.01]	0.96 [0.94–0.97]	0.03 [0.01–0.05]	0.00 [0.00–0.00]	0.00 [0.00–0.00]	0.97 [0.95–0.99]
**NH**	-	-	0.00 [0.00–0.00]	1.00 [1.00–1.00]	-	-	0.00 [0.00–0.00]	1.00 [1.00–1.00]

OH: Ordinary Home; IH: Intermediate Housing; NH: Nursing Home.

*from multi state model including 4 states (OH, IH, NH and death) controlled for sex.

From the analytic sample (N = 5237), 878 participants (16.7%) experienced a residential transition from OH to another type of housing (detailed in Supplementary materials, **Figure S1**).

### 3.3 Associations between disability and cognition with residential transitions

Higher levels of functional disability were significantly associated with an increased risk of transitioning from OH to IH or NH over time, after adjustment for potential confounders including cognition (Table 3). Compared with individuals without disability, those with mild or moderate disability had higher risks of transition from OH to IH (**HR=2.81, 95% CI= [1.71; 4.61] and HR=3.06, 95% CI= [1.79; 5.23],** respectively), while no association was found for severe disability. Risks of transitioning from OH to NH increased markedly with disability severity: **HR=2.53, 95% CI= [1.63; 3.93], HR=8.85, 95% CI= [5.73; 13.68]**, and **HR=14.00, 95% CI= [8.20; 23.07]**, for mild, moderate and severe disability respectively. No significant associations were found for other transitions, although association was suggested between the increased disability severity and the transition from IH to NH.

**Table 3:** Hazard ratios from the multi-state model: functional disability and cognitive performance, and residential transitions (Ordinary Home, Intermediate Housing and Nursing Home), from PAQUID (1988–2018), 3-City (1999–2018) and AMI (2007–2017) cohorts (N = 5237).

	HR [95% CI]^1^	*p-value*^2^	HR [95% CI]^1^	*p-value*^2^
From Ordinary Home →	To Intermediate Housing (N = 219)		To Nursing Home (N = 659)	
**Hierarchical disability**				
No	-	***<0.001***	-	***<0.001***
Mild	**2.81 [1.71; 4.61]**		**2.53 [1.63; 3.93]**	
Moderate	**3.06 [1.79; 5.23]**		**8.85 [5.73; 13.68]**	
Severe	1.53 [0.47; 4.95]		**14.00 [8.50; 23.07]**	
**MMSE Score −1**	**1.06 [1.01; 1.12]**	***0.029***	**1.11 [1.09; 1.14]**	***<0.001***
**From Intermediate Housing →**	**To Nursing Home** (N = 38)		**To Ordinary Home** (N = 40)	
**Hierarchical disability**				
No	-	*0.053*	-	*0.622*
Mild	1.06 [0.13; 8.61]		1.66 [0.48; 5.76]	
Moderate	1.66 [0.21; 13.03]		1.31 [0.37; 4.68]	
Severe	3.67 [0.42; 32.14]		0.81 [0.08; 8.31]	
**MMSE Score −1**	**1.13 [1.01; 1.22]**	***0.025***	0.98 [0.85; 1.13]	*0.806*

HR: Hazard ratio; CI: Confidence interval.

1 Multi state model adjusted for sex, cohort, marital status and educational level.

2 Global p-value of the log-likelihood ratio test.

Independently of disability, lower cognitive performance was associated with higher risks of transition. Each one-point decrease in MMSE score was associated with increased risk of transitioning from OH to IH (**HR=1.06, 95% CI= [1.01; 1.12]**), from OH to NH (**HR=1.11, 95% CI= [1.09–1.14]**), and from IH to NH (**HR=1.13, 95% CI= [1.01–1.22]**).

Sensitivity analyses using lagged functional disability and cognition (measured at the preceding visit) showed similar significant associations for the hierarchical disability indicator. Associations for MMSE were attenuated, and MMSE was no longer significantly associated with transitions from OH to IH or from IH to NH (Supplementary materials, **Table S2**). Moreover, additional adjustment for polypharmacy did not alter the main results (Supplementary materials, **Table S3**).

### 3.4 Cognitive impairment and residential transitions

Cognitive impairment (MMSE score≤ 23) was also associated with higher risks of transitioning from OH to NH (**HR=2.72, 95% CI= [2.23; 3.31]**), with no significant associations for other transitions (Supplementary materials, **Table S4**).

### 3.5 Combined association of IADL disability and cognitive impairment

Compared with individuals free of IADL and cognitive impairment, both IADL disability alone and cognitive impairment alone were associated with an increased risk of transitioning from OH to IH (**HR=1.94, 95% CI= [1.14; 3.28]** and **HR=1.82, 95% CI= [1.29–2.56]** respectively), while their simultaneous presence was not (Table 4). For transitions form OH to NH, risks rose sharply with combined impairment (**HR=16.31, 95% CI= [12.52–21.26]**), and remained elevated for those with IADL disability only (**HR=3.78, 95% CI= [2.57; 5.55]**) or cognitive impairment only (**HR=5.46, 95% CI= [4.31; 6.94]**).

**Table 4:** Hazard ratios from the multi-state model: combination of IADL disability and cognitive impairment*, and residential transitions (Ordinary Home, Intermediate Housing and Nursing Home), from PAQUID (1988–2018), 3-City (1999–2018) and AMI (2007–2017) cohorts (N = 5237).

	HR [95% CI]^1^	*p-value*^2^	HR [95% CI]^1^	*p-value*^2^
From Ordinary Home →	To Intermediate Housing (N = 219)		To Nursing Home (N = 659)	
No disability no cognitive impairment	-	***<0.001***	-	***<0.001***
Only IADL disability	**1.94 [1.14; 3.28]**		**3.78 [2.57; 5.55]**	
Only cognitive impairment	**1.82 [1.29; 2.56]**		**5.46 [4.31; 6.93]**	
IADL disability + cognitive impairment	0.92 [0.50; 1.70]		**16.31 [12.52; 21.26]**	
**From Intermediate Housing →**	**To Nursing Home** (N = 38)		**To Ordinary Home** (N = 40)	
No disability no cognitive impairment	-	*0.161*	-	*0.578*
Only IADL disability	1.49 [0.31; 7.04]		0.48 [0.07; 3.19]	
Only cognitive impairment	2.32 [0.87; 6.23]		0.89 [0.44; 1.79]	
IADL disability + cognitive impairment	2.84 [0.83; 9.65]		0.60 [0.16; 2.19]	

HR: Hazard ratio; CI: Confidence interval.

1 Multi state model adjusted for sex, cohort, marital status and educational level.

2 Global p-value of the log-likelihood ratio test * Cognitive impairment: MMSE Score ≤23/30.

### 3.6 Sex-specific associations with each IADL disability

After excluding missing IADL data, the final stratified samples comprised 2981 women and 2254 men, among whom 151 women and 46 men transitioned from OH to IH, and 476 women and 140 men transitioned from OH to NH during follow-up.

When exploring specific IADL items controlled for global IADL score (Table 5), notable sex differences emerged. In women, difficulties with transportation were significantly associated with transitions from OH to IH (**HR=1.67, 95% CI= [1.05; 2.64]**), and from OH to NH (**HR=1.74, 95% CI= [1.31; 2.32]**). Difficulties with shopping, handling finances and housekeeping were associated only with transition from OH to NH (**HR=2.37, 95% CI= [1.76; 3.18], HR=1.34, 95% CI= [1.00; 1.79]** and **HR=1.92, 95% CI= [1.47; 2.52]**, respectively). Some items, including handling finances and meal preparation, showed a trend toward lower risk of transition from OH to IH, whereas difficulties in using the telephone showed a trend toward a lower risk of transition from OH to NH (p-values<0.10). In men, no significant associations were found for OH to IH transition. However, difficulties with shopping, transportation and managing medications were associated with transition to NH (**HR=2.04, 95% CI= [1.17; 3.55], HR=2.23, 95% CI= [1.28; 3.88]** and **HR=1.78, 95% CI= [1.01; 3.14]**, respectively).

**Table 5:** Hazard ratios from the multi-state model for residential transitions (Ordinary Home, Intermediate Housing and Nursing Home) stratifying by sex, from PAQUID (1989–2019), 3-City (1999–2018) and AMI (2007–2017) cohorts (N = 5235*).

	Women (n = 2981)			
	HR [95% CI]^1^	*p-value*^2^	HR [95% CI]^1^	*p-value*^2^
From Ordinary Home →	To Intermediate Housing		To Nursing Home	
	*N=151*		*N=476*	
**IADL ability: need help / difficulty to**				
Use the telephone	0.55 [0.25; 1.20]	*0.133*	0.75 [0.56; 1.01]	*0.057*
Do the shopping	1.20 [0.72; 2.00]	*0.476*	**2.37 [1.76; 3.18]**	***<0.001***
Use transportation	**1.67 [1.05; 2.64]**	***0.029***	**1.74 [1.31; 2.32]**	***<0.001***
Manage medications	0.79 [0.28; 2.20]	*0.650*	1.19 [0.87; 1.64]	*0.279*
Handle finances	0.52 [0.27; 1.01]	*0.053*	**1.34 [1.00; 1.79]**	***0.050***
Meal preparation^⁎⁎^	0.50 [0.23; 1.11]	*0.089*	0.98 [0.71; 1.34]	*0.881*
Housekeeping^⁎⁎^	1.33 [0.89; 2.01]	*0.168*	**1.92 [1.47; 2.52]**	***<0.001***
Laundry^⁎⁎^	0.83 [0.41; 1.66]	*0.598*	1.01 [0.76; 1.36]	*0.928*
	**Men (n=2254)**			
	**HR [95% CI]**^1^	***p-value***^2^	**HR [95% CI]**^1^	***p-value***^2^
**From Ordinary Home →**	**To Intermediate Housing**		**To Nursing Home**	
	*N=46*		*N=140*	
**IADL ability: need help / difficulty to**				
Use the telephone	0.87 [0.18; 4.15]	*0.862*	1.17 [0.67; 2.06]	*0.582*
Do the shopping	2.41 [0.84; 6.95]	*0.102*	**2.04 [1.17; 3.55]**	***0.011***
Use transportation	1.13 [0.29; 4.41]	*0.857*	**2.23 [1.28; 3.88]**	***0.005***
Manage medications	0.65 [0.10; 4.33]	*0.661*	**1.78 [1.01; 3.14]**	***0.046***
Handle finances	0.36 [0.09; 1.38]	*0.135*	0.84 [0.47; 1.48]	*0.539*

HR: Hazard ratio; CI: Confidence interval.

1 Multi-state model adjusted for cohort, marital status, educational level, MMSE score and score for other IADL abilities.

2 P-value of the log-likelihood ratio test *Exclusion of 2 supplementary individuals without two complete visits due to missing data on IADL disability ^⁎⁎^ Only for women.

In lagged analysis (Supplementary materials, **Table S5**), results were generally attenuated. In women, housekeeping difficulties became significantly associated with OH to IH transition, while difficulties with shopping, transportation and housekeeping remained associated with OH to NH transition, and transportation difficulties showed a trend toward OH to IH transition (p-value=0.075). In men, only shopping and transportation remained significantly associated with OH to NH transition.

## 4 Discussion

This study explored the relationship between functional disability, cognition, and residential transitions among older adults from three French population-based cohorts. Over 30-years, 18.7% of participants moved from OH, mostly to NH (77.5%) and to less often to IH (22.5%). As expected, the probabilities of remaining at home declined sharply with age, with transitions to NH being more frequent than those to IH. Independently of marital status and cognition, mild or moderate functional disability (mobility and IADL disabilities), tripled the risk of transitioning from OH to IH compared with individuals free of disability, whereas severe disability was not associated. Risks of transitioning from OH to NH increased exponentially with disability severity, reaching a 14-fold higher risk among those severely disabled. Combined disability and cognitive decline further amplified the risk of entering NH (HR=16.31), while transitions from IH to NH were close to significance (HR=2.84, 95% CI= [0.83–9.65]). Transitions to intermediate housing were more frequently observed among individuals whose limitations did not critically affect daily functioning, whereas transitions to NH were more common in participants with advanced cognitive decline (with repercussions on daily living). Sex-specific analyses further revealed that only difficulty with transportation in women was associated with transition from OH to IH, whereas no significant association was reported in men for this transition. For the transition from OH to NH, difficulties with shopping and transportation were associated with increased risks in both sexes. Difficulty in handling finances was associated with NH admission only in women, while difficulty with medication management was associated only in men. When considering lagged IADL disability (measured at T-1), associations were generally slightly attenuated, suggesting that IADL disabilities may act as proximal triggers for residential transitions rather than long-term predictors.

Fewer than one in five participants changed residence, underscoring both the strong preference and potential structural barriers to remaining at home as long as possible, in line with surveys reporting that most older adults wish to age in place and avoid institutionalization [38], [39]. The 18.7% transition rate observed here is consistent with a previous study (Chyr et al. [22]) and supports the association between functional decline and relocation in senior housing, as previously reported [20,21,40,41].

Our study clarifies which specific functional limitations drive residential transitions. In PAQUID, shopping was previously identified as the first IADL lost in the dependency process [16], a finding echoed here for both sexes for NH admission. Similar associations between cooking or housekeeping difficulties, cognitive impairment, and relocation were reported by Grandbon et al. [24] and Boldy et al. [42], reinforcing the role of IADL decline (especially in household and mobility activities) in residential transitions. Together, these findings suggest a two-stage decline: an initial non-cognitive phase, characterized by mobility or domestic difficulties that could be partly compensated by IH services (housekeeping, laundry, transportation services, collective catering); and a later cognitive phase involving executive-function tasks (e.g. financial or medications management), which may precipitate admission to NH due to behavioral or safety concerns. This continuum highlights the progressive nature of dependency and the need for housing models adapted along the disablement trajectory. However, some sex-specific patterns were observed. Transportation difficulties were associated with all transitions in women and other sex-differences emerged for NH admission (difficulties with financial management in women and with medication management in men). These results may reflect gendered life-course role allocation in the studied generations, whereby certain activities carried distinct functional and symbolic meanings (e.g., men typically responsible for driving or handling finances, and women for managing medications).

Beyond functional and cognitive disabilities, the decision to relocate in later life is influenced by multiple contextual factors. Roy et al. identified six main dimensions shaping housing decisions [43]: socioeconomic and health factors, economic aspects, psychological and psychosocial factors, social relationship, the built and natural environment, and the time/space-time dimension. Our findings echo several of these inking mobility and domestic activities to spatial and temporal constraints, transport and service access to environmental factors, and family or financial support to the social domain. This confirms that functional decline interacts with contextual determinants in shaping residential trajectories [44].

The IADL-related transitions observed highlight potential targets for more proactive and personalized services (particularly in transportation) to help older adults remain at home longer. Although some associations differed by sex and were attenuated in lagged analyses, mobility and management related difficulties appeared to act as proximal markers of residential transition. Timely identification of those at risk remains a key challenge in order to intervene upstream in the disablement process. This approach aligns with the WHO’s Integrated Care for Older People (ICOPE) framework, which emphasizes early detection of decline across key domains (mobility, cognition, psychological health, vision, audition and vitality) and targeted interventions to maintain independency [45]. Integrating early, personalized, and sex-sensitive support could contribute to maintain living in place, optimize the use of IH resources, and ultimately may inform strategies to delay premature transitions to more medicalized settings such as NH. Yet, current societies face major challenges in providing adequate housing solutions for older adults. While policies increasingly encourage aging in place, it is not feasible for everyone, particularly for those with specific disabilities, health risks, or social isolation, for whom IH remains a valuable alternative. Further research is needed to explore how IH can be optimized as an intermediate step within a continuum of aging environments.

### 4.1 Strengths and limitations

This study benefits from its large sample (> 5000 participants), exceptionally long follow-up (up to 30 years), and the integration of both functional and cognitive measures. The use of multi-state models captured complex, time-dependent transitions and competing risks, while the sex-stratified analysis revealed novel gender-specific pathways. Limitations include the exclusive use of French data, which may limit generalizability, although the inclusion of both urban and rural participants enhances representativeness. In addition, the three cohorts span different historical periods (1988–2018), during which the availability and organization of intermediate housing options in France have evolved. Although models adjusted for cohort, some residual heterogeneity related to temporal or regional differences in care infrastructure cannot be excluded. It should also be noted that IADL assessment differs by sex in the Lawton scale, with men evaluated on five items and women on eight, reflecting traditional gender roles in these generations. This difference may influence the identification of early functional decline and the construction of the hierarchical disability indicator. However, analyses were stratified by sex and IADL items were examined individually, which partly mitigates this limitation. A small number of participants (n = 310) were excluded due to incomplete follow-up, and these individuals were generally older and in poorer health, which may have slightly underestimated transitions toward adverse health states (Supplementary Table S1). The small number of transitions from IH to NH or back to OH reduced power for these specific pathways, but the identification of subgroups (mainly women) experiencing return transitions highlights the need for adaptable housing solutions. Finally, data collection began in 1988, before major policy shifts promoting aging in place, but extended until 2018, offering a long-term perspective on residential trajectories. While this long-term perspective provides valuable insight, future research using more recent cohorts will be essential to account for evolving housing models and new digital-related instrumental activities (e.g. online services, digital banking) that may shape dependency trajectories. Further studies could also explore how the combined evolution of cognitive decline and functional limitations influences residential decision-making, and whether early identification of specific disability profiles could help anticipate housing transitions and support aging in place.

## 5 Conclusion

This study provides new insights into the complex relationship between functional disability, cognition, and residential transitions in older adults particularly via IH. Mild-to-moderate functional decline was more frequently followed by transitions to intermediate housing, while severe disability or cognitive impairment were associated with long-term care placement. Marked sex-specific differences in IADL limitations highlight the need for tailored support strategies. Early identification of these functional patterns may help anticipate residential transitions and inform strategies aimed at supporting aging in place and improving the organization of housing options for older adults.

## Funding sources

This work was supported by the Priority Research Program (PPR) Autonomy which received government funding managed by the French National Research Agency under France 2030, reference ANR-23-PAVH-0007.

## Declaration of generative AI and AI-assisted technologies in the manuscript preparation process

An AI language model (ChatGPT) was used exclusively for English language editing. After using this service, the authors reviewed and edited the content as needed and take(s) full responsibility for the content of the published article.

## Data statement

Data described in the manuscript, code book, and analytic code will be made available upon request: https://etudesactive.wixsite.com/etudes-active/.

## CRediT authorship contribution statement

**Jeanne Bardinet:** Writing – review & editing, Writing – original draft, Visualization, Validation, Software, Project administration, Methodology, Investigation, Formal analysis, Data curation, Conceptualization. **Luc Letenneur:** Writing – review & editing, Visualization, Validation, Project administration, Methodology. **Alice Pellichero:** Writing – review & editing, Visualization, Validation, Methodology. **Denis Boucaud-Maitre:** Writing – review & editing, Visualization, Validation, Conceptualization. **Maturin Tabue-Teguo:** Writing – review & editing, Visualization, Validation. **Hélène Amieva:** Writing – review & editing, Visualization, Validation, Project administration, Methodology. **Karine Pérès:** Writing – review & editing, Writing – original draft, Visualization, Validation, Supervision, Resources, Project administration, Methodology, Investigation, Funding acquisition, Conceptualization.

## Declaration of competing interest

The authors declare that they have no known competing financial interests or personal relationships that could have appeared to influence the work reported in this paper.

## Appendix Supplementary materials

Image, application 1
