# Supplementary material for: Functional disability and cognition in relation to residential transitions in older adults: Evidence from three French cohorts, 1988-2018
Source: J Frailty Aging. 2026 May 24;15(3):100159. doi: 10.1016/j.tjfa.2026.100159 (PMC13224349; doi:10.1016/j.tjfa.2026.100159)
Supplement: Supplementary file 1 [file mmc1.docx]

**Supplementary Materials**

**Figure S1.** Residential transitions of analytic sample of older adults living in OH (n=5,237)

**Table S1.** Transition intensity matrix (per year) for eligible (n=5,547) and for analytic sample (n=5,237)

**Table S2.** Hazard ratios from the lagged multi-state model: functional disability, cognitive performance, and residential transitions (n=5,346)

**Table S3.** Hazard ratios from the multi-state model: functional disability and cognitive performance, and residential transitions, additionally adjusted for polypharmacy (N=5,237)

**Table S4.** Hazard ratios from the multi-state model: functional disability, cognitive impairment and residential transitions (N=5,237)

**Table S5.** Hazard ratios from the lagged multi-state model for residential transitions stratifying by sex (N=5,346)

**Figure S1.** Residential transitions of **analytic sample** of older adults living in OH, from PAQUID, 3C and AMI cohorts, 1988-2018 (n=5,237)

Intermediate Housing

**Ordinary Home**

**N=5,237**

Nursing Home

N=659

N=219

N=40

N=38

**Table S1.** Transition intensity matrix (per year) for eligible (n=5,547) and for analytic sample (n=5,237), from PAQUID (1989-2019), 3-City (1999-2018) and AMI (2007-2017) cohorts.

|  | **Eligible Sample**  **(N=5,547)** | | | | |  |  |  |  |
| --- | --- | --- | --- | --- | --- | --- | --- | --- | --- |
| **To** | *Mean sojourn time* | **OH** | **IH** | **NH** | **Death** |  |  |  |  |
| **From** | *(in year)* |  | | | |  |  |  |  |
| **OH** | 12.42 [12.07 ; 12.78] | -0.080 [-0.083 ; -0.078] | 0.006 [0.005 ; 0.007] | 0.019 [0.017 ; 0.020] | 0.056 [0.054 ; 0.058] |  |  |  |  |
| **IH** | 3.85 [3.37 ; 4.41] | 0.052 [0.038-0.072] | -0.259 [-0.297 ; -0.227] | 0.097 [0.073 ; 0.128] | 0.110 [0.087 ; 0.138] |  |  |  |  |
| **NH** | 3.10 [2.89 ; 3.33] | - | - | -0.322 [-0 .346 ; -0.300] | 0.322 [0.300 ; 0.346] |  |  |  |  |
|  | **Analytic Sample**  **(N=5,237)** | | | | |  |  |  |  |
| **To** | *Mean sojourn time* | **OH** | **IH** | **NH** | **Death** |  |  |  |  |
| **From** | *(in year)* |  |  |  |  |  |  |  |  |
| **OH** | 13.27 [12.87 ; 13.68] | -0.075 [-0.078 ; -0.073] | 0.006 [0.005 ; 0.007] | 0.016 [0.015 ; 0.018] | 0.053 [0.051 ; 0.055] |  |  |  |  |
| **IH** | 3.91 [3.38 ; 4.52] | 0.057 [0.041-0.079] | -0.256 [-0.296 ; -0.221] | 0.089 [0.064 ; 0.122] | 0.111 [0.087 ; 0.141] |  |  |  |  |
| **NH** | 3.21 [2.94 ; 3.49] | - | - | -0.312 [-0 .340 ; -0.286] | 0.312 [0.286 ; 0.340] |  |  |  |  |

OH: Ordinary Home; IH: Intermediate Housing; NH: Nursing Home

Values are maximum likelihood estimates with 95% confidence intervals in parentheses.
Diagonal elements represent the total rate of leaving each state ($q_{ii}$= -$\sum q_{ij}$).
Dashes (-) indicate structurally constrained zero transitions.

**Table S2.** Hazard ratios from the **lagged** multi-state model: functional disability, cognitive performance, and residential transitions (Ordinary Home, Intermediate Housing and Nursing Home), from PAQUID (1988-2018), 3-City (1999-2018) and AMI (2007-2017) cohorts (N=5,346)

|  | **HR [95% CI]^1^** | ***p-value^2^*** | **HR [95% CI]^1^** | ***p-value^2^*** |
| --- | --- | --- | --- | --- |
| **From Ordinary Home →** | **To Intermediate Housing**  (N=221) |  | **To Nursing Home**  (N=741) |  |
| **Hierarchical disability** |  |  |  |  |
| No | - | ***<0.001*** | - | ***<0.001*** |
| Mild | **2.45 [1.61 ; 3.72]** |  | **2.69 [1.99 ; 3.64]** |  |
| Moderate | **2.52 [1.55 ; 4.08]** |  | **6.81 [5.01 ; 9.25]** |  |
| Severe | 1.10 [0.21 ; 5.79] |  | **7.44 [4.67 ; 11.86]** |  |
| **MMSE Score -1** | 1.06 [0.99 ; 1.12] | *0.061* | **1.09 [1.07 ; 1.12]** | ***<0.001*** |
| **From Intermediate Housing →** | **To Nursing Home**  (N=49) |  | **To Ordinary Home**  (N=40) |  |
| **Hierarchical disability** |  |  |  |  |
| No | - | ***0.014*** | - | *0.117* |
| Mild | 1.42 [0.26 ; 7.92] |  | 1.04 [0.42 ; 2.58] |  |
| Moderate | 2.77 [0.50 ; 15.30] |  | 0.53 [0.17 ; 1.63] |  |
| Severe | 3.03 [0.26 ; 35.24] |  | 1.52 [0.13 ; 15.80] |  |
| **MMSE Score -1** | 1.09 [0.98 ; 1.21] | *0.109* | 1.04 [0.90 ; 1.20] | *0.567* |

HR: Hazard ratio; CI: Confidence interval

^1^ Multi state model adjusted for sex, cohort, marital status and educational level

^2^ Global p-value of the log-likelihood ratio test

**Table S3.** Hazard ratios from the multi-state model: functional disability and cognitive performance, and residential transitions (Ordinary Home, Intermediate Housing and Nursing Home), additionally adjusted for polypharmacy, from PAQUID (1988-2018), 3-City (1999-2018) and AMI (2007-2017) cohorts (N=5,237)

|  | **HR [95% CI]^1^** | ***p-value^2^*** | **HR [95% CI]^1^** | ***p-value^2^*** |
| --- | --- | --- | --- | --- |
| **From Ordinary Home →** | **To Intermediate Housing**  (N=219) |  | **To Nursing Home**  (N=659) |  |
| **Hierarchical disability** |  |  |  |  |
| No | - | ***<0.001*** | - | ***<0.001*** |
| Mild | **2.59 [1.59 ; 4.22]** |  | **2.42 [1.56 ; 3.75]** |  |
| Moderate | **2.64 [1.54 ; 4.52]** |  | **8.26 [5.34 ; 12.78]** |  |
| Severe | 1.41 [0.45 ; 4.42] |  | **13.05 [7.89 ; 21.60]** |  |
| **MMSE Score -1** | **1.06 [1.00 ; 1.12]** | ***0.035*** | **1.11 [1.09 ; 1.14]** | ***<0.001*** |
| **From Intermediate Housing →** | **To Nursing Home**  (N=38) |  | **To Ordinary Home**  (N=40) |  |
| **Hierarchical disability** |  |  |  |  |
| No | - | ***0.048*** | - | *0.812* |
| Mild | 1.30 [0.13 ; 13.23] |  | 1.80 [0.51 ; 6.40] |  |
| Moderate | 2.05 [0.21 ; 20.21] |  | 1.60 [0.43 ; 5.90] |  |
| Severe | 4.95 [0.42 ; 58.57] |  | 1.04 [0.08 ; 12.67] |  |
| **MMSE Score -1** | **1.13 [1.05 ; 1.22]** | ***0.033*** | 0.99 [0.86 ; 1.14] | *0.861* |

HR: Hazard ratio; CI: Confidence interval

^1^ Multi state model adjusted for sex, cohort, marital status, educational level and **polypharmacy***

^2^ Global p-value of the log-likelihood ratio test

*Polypharmacy = ≥6 medications

**Table S4.** Hazard ratios from the multi-state model: functional disability, **cognitive impairment*** and residential transitions (Ordinary Home, Intermediate Housing and Nursing Home), from PAQUID (1988-2018), 3-City (1999-2018) and AMI (2007-2017) cohorts (N=5,237)

|  | **HR [95% CI]^1^** | ***p-value^2^*** | **HR [95% CI]^1^** | ***p-value^2^*** |
| --- | --- | --- | --- | --- |
| **From Ordinary Home →** | **To Intermediate Housing**  (N=219) |  | **To Nursing Home**  (N=659) |  |
| **Hierarchical disability** |  |  |  |  |
| No | - | ***<0.001*** | - | ***<0.001*** |
| Mild | **3.09 [1.90 ; 5.05]** |  | **2.72 [2.23 ; 3.31]** |  |
| Moderate | **3.66 [2.16 ; 6.20]** |  | **2.48 [1.62 ; 3.79]** |  |
| Severe | 2.45 [0.83 ; 7.21] |  | **8.81 [5.79 ; 13.42]** |  |
| **Cognitive impairment*** | 0.96 [0.63 ; 1.48] | *0.869* | **2.72 [2.23 ; 3.31]** | ***<0.001*** |
| **From Intermediate Housing →** | **To Nursing Home**  (N=38) |  | **To Ordinary Home**  (N=40) |  |
| **Hierarchical disability** |  |  |  |  |
| No | - | *0.062* | - | *0.900* |
| Mild | 0.99 [0.12 ; 8.15] |  | 1.90 [0.53 ; 6.77] |  |
| Moderate | 1.86 [0.24 ; 14.22] |  | 1.55 [0.43 ; 5.65] |  |
| Severe | 5.91 [0.66 ; 52.55] |  | 0.97 [0.07 ; 14.34] |  |
| **Cognitive impairment*** | 1.29 [0.54 ; 3.05] | *0.566* | 0.44 [0.13 ; 1.48] | *0.186* |

HR: Hazard ratio; CI: Confidence interval

^1^ Multi state model adjusted for sex, cohort, marital status and educational level

^2^ Global p-value of the log-likelihood ratio test

*Cognitive impairment: MMSE score≤23/30

In multi-state model adjusted for sex, cohort, marital status, educational level and hierarchical disability, cognitive impairment (MMSE score≤ 23) was associated with higher risks of transitioning from OH to NH (**HR=2.72, 95% CI=[2.23-3.31]**), with no significant associations for other transitions.

**Table S5.** Hazard ratios from the **lagged** multi-state model for residential transitions (Ordinary Home, Intermediate Housing and Nursing Home) stratifying by sex, from PAQUID (1989-2019), 3-City (1999-2018) and AMI (2007-2017) cohorts (N=5,346)

|  | **Women (n=3,042)** | | | |
| --- | --- | --- | --- | --- |
|  | **HR [95% CI]^1^** | ***p-value^2^*** | **HR [95% CI]^1^** | ***p-value^2^*** |
| **From Ordinary Home →** | **To Intermediate Housing** |  | **To Nursing Home** |  |
|  | *N=157* |  | *N=574* |  |
| **IADL ability: need help / difficulty to** | | | | |
| Use the telephone | 0.41 [0.15 ; 1.09] | *0.073* | 0.79 [0.58 ; 1.08] | *0.142* |
| Do the shopping | 0.96 [0.55 ; 1.70] | *0.892* | **2.10 [1.61 ; 2.75]** | ***<0.001*** |
| Use transportation | 1.54 [0.96 ; 2.48] | *0.075* | **2.56 [1.98 ; 3.31]** | ***<0.001*** |
| Manage medications | 0.54 [0.14 ; 1.94] | *0.342* | 0.84 [0.59 ; 1.18] | *0.316* |
| Handle finances | 0.93 [0.46 ; 1.89] | *0.850* | 1.16 [0.86 ; 1.56] | *0.339* |
| Meal preparation* | 0.53 [0.21 ; 1.31] | *0.168* | 0.81 [0.59 ; 1.10] | *0.182* |
| Housekeeping* | **1.98 [1.33 ; 2.94]** | ***<0.001*** | **2.24 [1.77 ; 2.84]** | ***<0.001*** |
| Laundry* | 0.68 [0.32 ; 1.45] | *0.318* | 0.79 [0.59 ; 1.06] | *0.123* |
|  | **Men (n=2,302)** | | | |
|  | **HR [95% CI]^1^** | ***p-value^2^*** | **HR [95% CI]^1^** | ***p-value^2^*** |
| **From Ordinary Home →** | **To Intermediate Housing** |  | **To Nursing Home** |  |
|  | *N=46* |  | *N=160* |  |
| **IADL ability: need help / difficulty to** | | | | |
| Use the telephone | 0.85 [0.19 ; 3.79] | *0.831* | 0.69 [0.37 ; 1.30] | *0.255* |
| Do the shopping | 1.07 [0.30 ; 3.80] | *0.916* | **1.98 [1.16 ; 3.39]** | ***0.012*** |
| Use transportation | 1.57 [0.40 ; 6.12] | *0.513* | **2.27 [1.25 ; 4.09]** | ***0.007*** |
| Manage medications | 0.81 [0.08 ; 8.68] | *0.864* | 1.32 [0.66 ; 2.62] | *0.427* |
| Handle finances | 0.54 [0.14 ; 2.08] | *0.370* | 0.77 [0.43 ; 2.08] | *0.377* |

HR: Hazard ratio; CI: Confidence interval

^1^ Multi-state model adjusted for cohort, marital status, educational level, MMSE score and score for other IADL abilities

^2^ P-value of the log-likelihood ratio test

* Only for women
